# An explainable machine learning model for predicting the outcome of ischemic stroke after mechanical thrombectomy

**DOI:** 10.1136/jnis-2022-019598

**Published:** 2022-11-29

**Authors:** Zhelv Yao, Chenglu Mao, Zhihong Ke, Yun Xu

**Affiliations:** 1 Department of Neurology, Nanjing University Medical School Affiliated Nanjing Drum Tower Hospital, Nanjing, Jiangsu, China; 2 Jiangsu Key Laboratory for Molecular Medicine, Nanjing University Medical School, Nanjing, China; 3 Nanjing Medicine Center For Neurological Diseases, Nanjing, China; 4 Department of Neurology, Nanjing Drum Tower Hospital, Clinical College of Nanjing Medical University, Nanjing, China

**Keywords:** Blood Flow, Intervention, Stroke, Thrombectomy

## Abstract

**Background:**

There is high variability in the clinical outcomes of patients with acute ischemic stroke (AIS) after mechanical thrombectomy (MT).

**Methods:**

217 consecutive patients with anterior circulation large vessel occlusion who underwent MT between August 2018 and January 2022 were analysed. The primary outcome was functional independence defined as a modified Rankin Scale score of 0–2 at 3 months. In the derivation cohort (August 2018 to December 2020), 7 ensemble ML models were trained on 70% of patients and tested on the remaining 30%. The model’s performance was further validated on the temporal validation cohort (January 2021 to January 2022). The SHapley Additive exPlanations (SHAP) framework was applied to interpret the prediction model.

**Results:**

Derivation analyses generated a 9-item score (PFCML-MT) comprising age, National Institutes of Health Stroke Scale score, collateral status, and postoperative laboratory indices (albumin-to-globulin ratio, estimated glomerular filtration rate, blood neutrophil count, C-reactive protein, albumin and serum glucose levels). The area under the curve was 0.87 for the test set and 0.84 for the temporal validation cohort. SHAP analysis further determined the thresholds for the top continuous features. This model has been translated into an online calculator that is freely available to the public (https://zhelvyao-123-60-sial5s.streamlitapp.com).

**Conclusions:**

Using ML and readily available features, we developed an ML model that can potentially be used in clinical practice to generate real-time, accurate predictions of the outcome of patients with AIS treated with MT.

**WHAT IS ALREADY KNOWN ON THIS TOPIC**

- Despite high rates of successful recanalization, a significant proportion of patients with acute ischemic stroke (AIS) have unfavorable outcomes after mechanical thrombectomy (MT).
- A more precise prediction tool to refine the prediction of AIS prognosis following MT, and identifying modifiable factors to further improve prognosis are still needed.

**WHAT THIS STUDY ADDS**

- We generated and validated the PFCML-MT score, a novel machine learning model, to predict clinical outcomes in patients with AIS undergoing MT. The model integrated nine easily accessible variables (age, National Institutes of Health Stroke Scale score, collateral status, and postoperative laboratory indices comprising albumin-to-globulin ratio, estimated glomerular filtration rate, blood neutrophil count, C-reactive protein, albumin and serum glucose levels), showed good discriminative performance, and was implemented in an online calculator, allowing patient-specific predictions and tailored therapeutic decisions.

**HOW THIS STUDY MIGHT AFFECT RESEARCH, PRACTICE OR POLICY**

- Accurate prognosis prediction can enable better clinical decision-making and improved information for patients, paving the way for more individualized approaches in stroke treatment. Future prospective clinical studies may elucidate the role of this model’s modifiable factors in further improving patients’ outcomes.

## Introduction

Mechanical thrombectomy (MT) is a vital component in the management of patients with acute ischemic stroke (AIS) with large vessel occlusion.^1^ Despite rates of successful recanalization up to 85%, not everyone benefits equally, and disability rates remain as high as 50–70% among these patients.^2^ The realization of this variability in outcomes has propelled efforts in the prognosis prediction of patients receiving MT. A precise prediction model for identifying outcomes could be valuable to both patients and clinicians by assisting medical staff in optimizing the postoperative treatment strategy and planning for the appropriate allocation of limited resources.

Prior studies aimed to predict outcomes after MT using conventional statistical approaches, which have limitations in extracting predictive markers from high-dimensional data and always yield suboptimal prediction performance.^3^ Moreover, most of the time windows in existing models have been restricted to 6–8 hours, and very few models have been built based on 24-hour time windows.^4^ With the time window extended to 24 hours, more precise and personalized models corresponding to current guidelines are urgently required.^5^ In addition, there remains a need to identify predictors that may affect the outcomes, especially those that are potentially modifiable.

In recent years, advances in computation and software technologies have contributed to machine learning (ML), which is optimized to learn from large amounts of high-dimensional data, fit data in a more flexible mathematical way, and provide a more precise prediction of outcomes.^6^ A small number of ML models have been established and yield good prediction performance.^7 8^ However, the complexity of ML models makes them difficult to interpret, and technical defects in balancing the complexity and accuracy of the model versus uncomplicated use largely hamper their clinical implementation.^9^

Herein, we developed and validated an ML-based model to predict outcome in patients with AIS receiving MT. We further explored the contributions of individual predictors to reflect the underlying decision path and developed a simple, yet effective, online calculator to increase the clinical translational value.

## Methods

### Study population

We retrospectively reviewed adult patients who received MT for large vessel occlusions of anterior circulation within 24 hours of symptom onset at our stroke center between August 1, 2018, and January 1, 2022. The exclusion criteria were patients with a pre-stroke modified Rankin Scale (mRS) score >2, combined posterior circulation ischemia, and missing data on 3-month clinical outcomes following MT. This research was approved by the ethics committee of Nanjing Drum Tower Hospital, and the requirement for individual informed consent was waived because of the study's retrospective nature.

### Data collection

Records in the database from three time points (preoperative, intraoperative, and within 1 day postoperatively) were used as early predictors. The variables comprised a range of domains, including demographic characteristics, clinical factors, laboratory indices, and radiological data.

### Study outcomes

Our main outcome was functional status, which was evaluated at 3 months by experienced staff through phone interviews using a mRS questionnaire. mRS scores of 0–2 and >2 were defined as good (ie, functional independence) and poor outcomes, respectively.

### ML analysis

Considering the weakness of instability and high risk of overfitting in a single classifier, we chose ensemble ML models to provide better prediction.^10^ For comparison, seven popular and up-to-date ensemble ML models—namely, the random forest (RF), gradient boosting (GB), eXtreme gradient boosting (XGBoost), categorical boosting (CatBoost), adaptive boosting (AdaBoost), light gradient boosting machine (LightGBM), and extra trees (ET) models, were established.

The ML analysis procedure involved five main steps: feature selection, model development, evaluation, interpretation, and deployment, and the specific process is displayed in online supplemental figure 1. All ML modeling and interpretation was conducted using Python 3 with the PyCaret library (version 2.3.10), scikit-Learn library (version 0.23.2), Streamlit (version 1.8.0), and SHapley Additive exPlanations (SHAP) module (version 0.40.0).

10.1136/jnis-2022-019598.supp1 Supplementary data

### Feature selection

First, we excluded potentially difficult-to-obtain variables. Subsequently, a systematic review of the relevant literature was performed to identify potential factors that need to be considered in the prediction of the outcome following MT. In total, 84 variables were selected as candidate predictors for the initial ML analysis on the basis of empirical observations. As listed in online supplemental table 1, the candidate predictors comprised two demographics, 10 comorbidities at admission, 54 laboratory values, nine radiological values, four stroke characteristics, and five treatment parameters. All potential candidate predictors were available in routine clinical settings.

### Model development

The eligible patients were divided into two groups: those who received MT between August 1, 2018 and December 31, 2020 (derivation cohort) and those who underwent MT between January 1, 2021 and January 1, 2022 (temporal validation cohort). Temporal validation is considered an in-between validation of internal and external validation.^11^

The derivation cohort was further randomly divided into two parts: the training set accounted for 70% and the test set for 30%. Models were trained with 10-fold cross-validation on the training set, and Bayesian optimization was applied to tune the hyperparameters of each ML algorithm. The hyperparameters that generated the largest area under the receiver operator characteristic curve (AUC) were chosen. See online supplemental table 2 for details of the selected hyperparameter values for each ML algorithm.

The model with the highest AUC in the test set was selected for further analysis. To minimize overfitting and facilitate use in clinical practice, we selected the least number of features to create the model while ensuring its performance. The features were successively deliminated according to the SHAP values until the AUC decreased significantly or until the reduction of other model evaluation indexes >0.05, and the final model is referenced as the PFCML-MT (personalized Prediction of outcome using Machine learnIng in patients undergoing MT) score throughout the manuscript.

### Model evaluation

The performance of the PFCML-MT score was evaluated using AUC, accuracy, sensitivity (recall), precision, and F1 score in the test set. To determine whether our model remained accurate when new data were entered, we further tested it on the temporal validation cohort.

### Model interpretation and deployment

Unlike regression or single decision tree models, ensemble ML classifiers do not generate regression coefficients or decision paths to facilitate the direct interpretation of complex models, so they are sometimes regarded as ‘black boxes’. SHAP analysis is preferred over other explainability methods because of local accuracy, consistency, and the ability to deal with missing values and was thus performed to gain insight into our model.^12^ In the present study, the impact and interaction among the predictors were explored by visualizing the SHAP values in global (ie, cohort level) and local (ie, patient-specific) forms.

Using the PFCML-MT score for prediction and the SHAP analysis for interpretation, we developed an online calculator in the Streamlit Python-based framework to facilitate clinical use.

### Statistical analysis

Continuous variables are expressed as the mean or median and were compared by the Student’s t test or the Mann‒Whitney U test, while categorical variables were presented as quantities and percentages and were compared using a Χ^2^ test or Fisher’s exact test, as appropriate. The AUC comparison was conducted using the DeLong test. All conventional statistical analyses were performed using SPSS version 25.0 (IBM SPSS, Inc., Chicago, Illinois, USA) and MedCalc version 20.022 (Ostend, Belgium). A two-sided P value <0.05 was considered statistically significant.

## Results

### Baseline characteristics

A total of 217 patients were eligible for the present study. Among them, 163 (75%) patients and 54 (25%) patients were allocated to the derivation and temporal validation cohorts, respectively. The main clinical characteristics of the derivation and temporal validation cohorts are detailed in online supplemental table 3. During the 3-month follow-up period, 52 (32%) patients achieved functional independence in the derivation cohort, and 20 (37%) acquired functional independence in the temporal validation cohort.

### Model development

The AUCs of the base ML models ranged from 0.83 to 0.90 in the test set (table 1). The RF classifier outperformed other ML models with the greatest AUC of 0.90 and was thus selected for all downstream analyses.

**Table 1:** Performance of machine learning models

Model		AUC	Recall	Accuracy	F1 score	Precision
Base model						
AdaBoost		0.83	0.80	0.73	0.65	0.55
LightGBM		0.88	0.67	0.82	0.69	0.71
XGBoost		0.88	0.93	0.76	0.70	0.56
Gradient Boosting		0.85	0.80	0.82	0.73	0.67
Extra trees		0.86	0.80	0.76	0.67	0.57
Random forest		0.90	0.80	0.82	0.73	0.67
CatBoost		0.89	0.80	0.80	0.71	0.63
Final model						
PFCML-MT	Test set	0.87	0.80	0.82	0.73	0.67
	Temporal validation set	0.84	0.75	0.78	0.71	0.68

AdaBoost, adaptive boosting; AUC, area under the receiver operator characteristic curve; CatBoost, categorical boosting; LightGBM, light gradient boosting machine; XGBoost, eXtreme gradient boosting.

The AUCs of the model with unrestricted predictors and the models restricted to 10, 20, or 30 predictors according to the SHAP values (online supplemental figure 2) are displayed in online supplemental figure 3. Given that the AUC of the 10-predictor model was not significantly lower than that of the others (DeLong test, all P>0.05), we focused on it that model further analysis. The final prediction model derived from the RF algorithm for clinical outcome selected through the SHAP method included nine features: age, National Institutes of Health Stroke Scale (NIHSS) score, collateral status, and postoperative laboratory indices (albumin-to-globulin ratio (AGR), albumin, estimated glomerular filtration rate (eGFR), blood neutrophil count, C-reactive protein (CRP), and serum glucose level).

### Model performance

**Figure 1:**
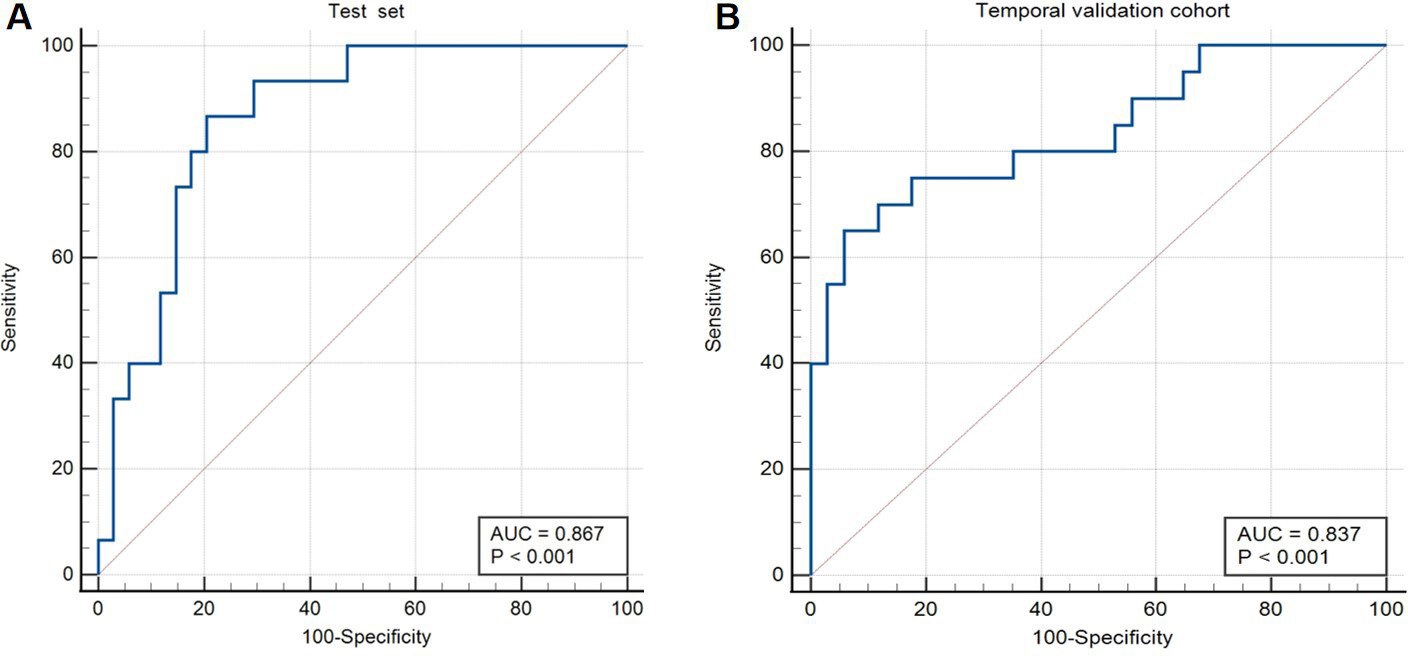
A,B Receiver operating characteristic curve analysis for the area under the curve (AUC) of the PFCML-MT model in the (A) test set and (B) temporal validation cohort.

The PFCML-MT score yielded high discrimination performance for predicting the outcome of patients with AIS who underwent MT, with an AUC of 0.87 (figure 1A), sensitivity of 0.80, accuracy of 0.82, F1 score of 0.73, and precision of 0.67 in the test set (table 1).

Validation of the PFCML-MT score in the temporal validation cohort generated consistent discrimination, with an AUC of 0.84 (figure 1B), sensitivity of 0.75, accuracy of 0.78, F1 score of 0.71, and precision of 0.68 (table 1), suggesting that our model is relatively reliable and stable.

### Model interpretation

**Figure 2:**
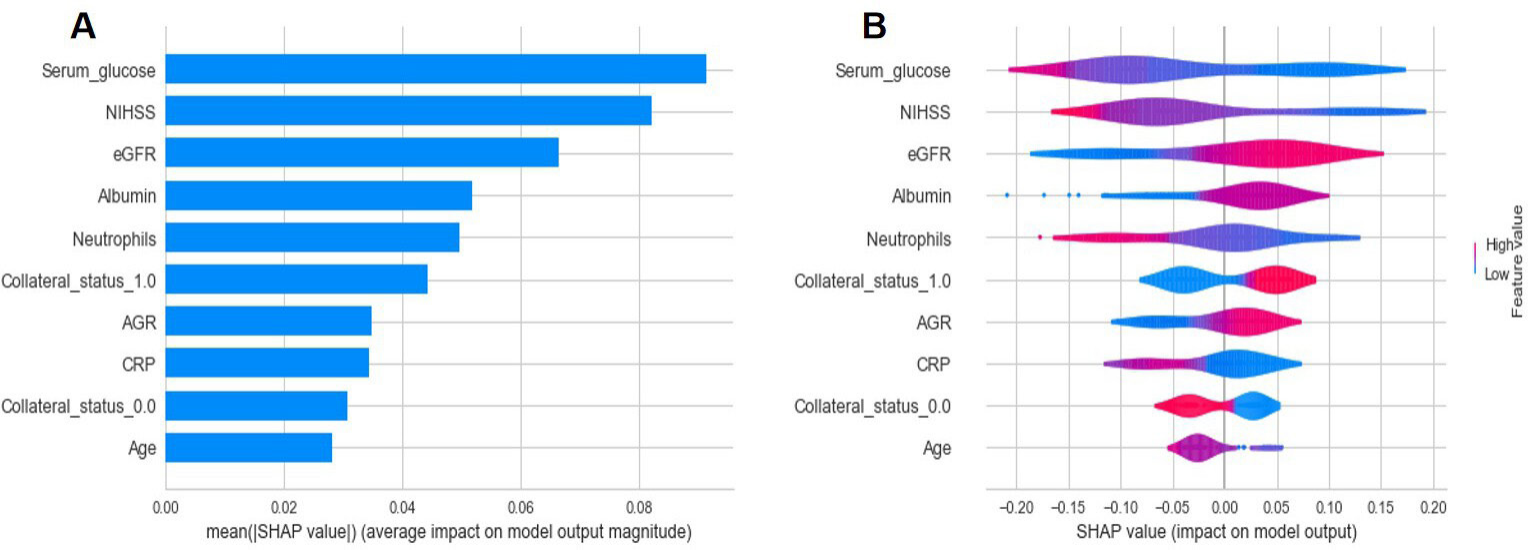
A,B SHAP summary plot of the impact of the features on the prediction of the PFCML-MT model. (A) Bar chart of the average SHAP value for each predictor. (B) Violin plots. The SHAP values (x-axis) represent the contribution of each predictor (y-axis). Features are ordered according to importance. Collateral status is a categorical variable and was preprocessed with one-hot encoding, thus its SHAP value is presented as ‘collateral_status_1.0’ and ‘collateral_status_0.0’ and its contribution is the sum. AGR, albumin-to-globulin ratio; CRP, C-reactive protein; eGFR, estimated glomerular filtration rate; NIHSS, National Institutes of Health Stroke Scale; SHAP, SHapley Additive exPlanations.

Figure 2A,B show the nine features contributing to the model in descending order, assessed by the average absolute SHAP values. As observed in the plot, serum glucose level, followed by NIHSS score, collateral status, eGFR, albumin level, blood neutrophil count, AGR, CRP, and age, were the nine most important predictors in the final model.

The SHAP dependence plot can also facilitate understanding of how an individual feature affects the output of the prediction model. Online supplemental figure 4A–H presents the SHAP values versus the measured value of each feature for the top continuous features. We can visualize how a feature’s attributed importance varies as its values change in the plot. For each predictor, a threshold can be determined from the figure to distinguish between a decreased risk (ie, SHAP value <0) and an increased risk (ie, SHAP value >0). For instance, an increase in the NIHSS score above 12 decreases the SHAP values and hence the odds of functional independence.

We also employed the SHAP method to interpret how the model makes personalized predictions for each specific instance. Online supplemental figure 5A displays a representative subject with functional independence whose outcome was correctly predicted by the model: the predicted probability of a good outcome was 77.32%. Online supplemental figure 5B shows a correctly predicted poor outcome for an individual, with a probability of 4.08%.

### Model deployment

**Figure 3:**
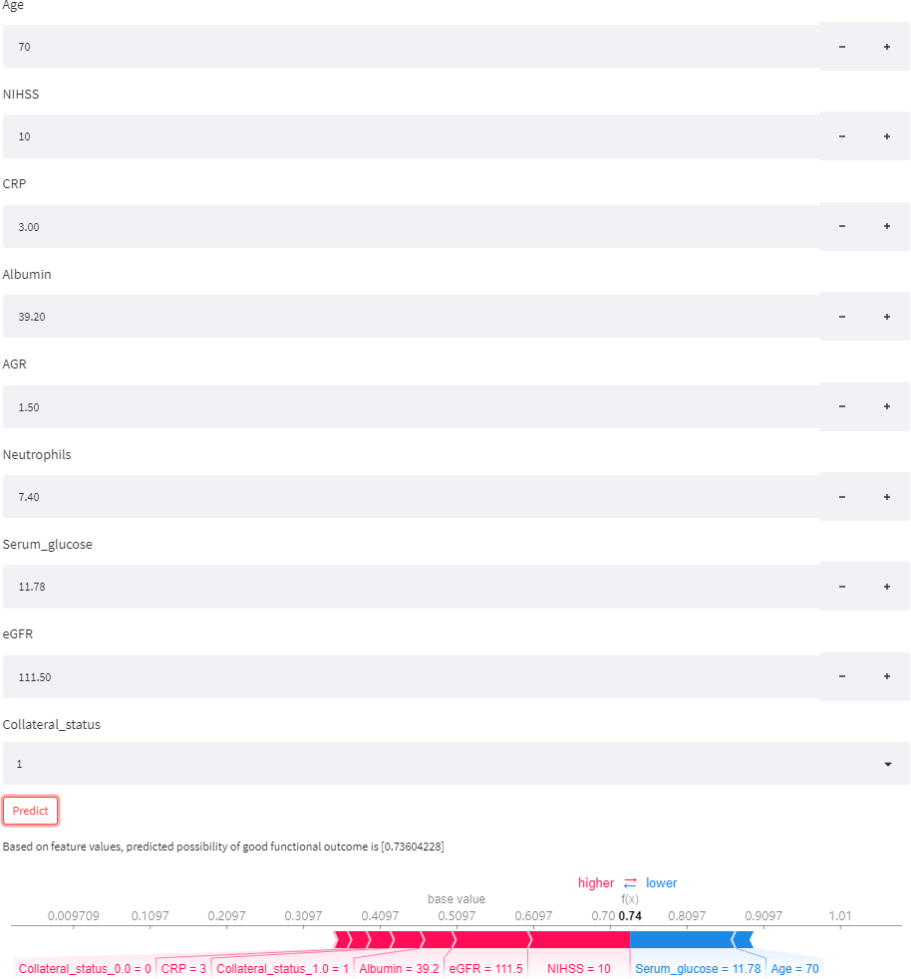
An example of online calculator use. By inputting the example values of nine required features, we can obtain a patient’s possibility of good outcome and an explanation of the prediction. AGR, albumin-to-globulin ratio; CRP, C-reactive protein; eGFR, estimated glomerular filtration rate; NIHSS, National Institutes of Health Stroke Scale.

A web-based version of the PFCML-MT model has been made available (https://zhelvyao-123-60-sial5s.streamlitapp.com) to allow for widespread use of the prediction tool (figure 3). This tool will automatically predict the outcome for patients with AIS who underwent MT when the values of the nine features required for the model are entered. Moreover, the online calculator provides users with the explanation of the prediction of the model, supports batch prediction of the outcome of multiple patients at one time, and can predict the outcome of patients with missing values.

## Discussion

Using explainable ML techniques, we identified a core set of predictors, determined their thresholds, and created a prognosis prediction model in patients with AIS treated with MT. The results indicate that the PFCML-MT model had high discriminatory power with an AUC of 0.87 in the test set and an AUC of 0.84 in the temporal validation cohort. All predictors came from routine clinical variables and corresponding easy-to-use online calculators, so this model could easily be applied to patients with AIS to improve clinical decision-making.

Some of the predictors selected in the model have provided us with novel insights. Although several predictors have been identified, such as age, NIHSS score, and preoperative glucose level,^13 14^ postoperative laboratory indices, comprising serum glucose level and indictors of liver function (AGR, albumin), renal function (eGFR), and inflammation (blood neutrophil count, CRP), were strong predictors that have been neglected by existing prediction models.^3–15^ In our study, we found that the predictive ability of postoperative laboratory indices outperformed that of preoperative indices and accounted for over half of the selected features in our model, highlighting the significant effects of postoperative conditions on outcomes following MT and suggesting that it should be evaluated in future studies. These factors are all from frequently recorded blood biochemistry tests or routine blood tests and, more importantly, are potentially modifiable. We further determined the threshold values at which these variables are critical for the patient’s prognosis with the hope of facilitating interventions to reduce disability and improve outcomes. Of note, the identified factor is not necessarily causal in explaining the prognosis. Further prospective interventional studies are warranted to evaluate the effect of modifiable factors and to identify additional modifiable factors.

In our study, we found that CT perfusion (CTP) parameters, consisting of perfusion defect (cerebral blood flow) < 30%, time to maximum (Tmax) > 6 s, Tmax >8 s, Tmax>10 s lesion), and mismatch ratio, significantly differed between the good and poor outcome groups and had predictive power but were not sufficient to be selected, while CT angiography (CTA)-based collateral status ranked as one of the most important predictors in the final model. Furthermore, as CTP imaging and its corresponding postprocessing software are not available in many centers across the globe, and inconsistency exists in various CTP postprocessing methods, the use of CTA may represent a more reasonable option for prognosis prediction. Some well-established predictors were not included in our model, such as onset-to-puncture time, potentially owing to the unknown onset time in some patients, such as patients with wake-up stroke, which represents approximately 20% of strokes.^16^ ^17^ Using the last known well time cannot represent the real onset time in these patients, thus partially obscuring the relationship between the time and prognosis. However, it also improves the generalization of our model, as we can predict the outcome of patients with unknown onset times. The 3-month functional independence rate in our derivation and temporal validation cohorts was slightly lower than in previous studies, which might be due to the inclusion of patients who underwent MT in the extended time window, and differences in treatment techniques and patients’ characteristics.

Several striking strengths distinguish this proposed prediction model from others reported previously for patients with AIS. First, robust and rigorous variable selection was conducted using ML. Our model was derived from a large initial pool of patient characteristics that were finally incorporated as the strongest predictors to identify outcomes and included nine clinical features, which are all routine clinical characteristics, as well as laboratory and radiological results that are available at most hospitals. In contrast, most established prediction models fail to fully consider variables at different time points (ie, preoperative, intraoperative, and postoperative) and different dimensions (ie, clinical, laboratory, and radiological), which might miss unexpected relationships and reduce the available information that could be used to improve the predictive power.^3^ In addition, our model has a certain tolerance for missing data, since we still achieved high performance on the temporal validation cohort for 10% cases with missing data, ensuring generalizability across different clinical settings. In real-world practice, missing data for some variables are inevitable, particularly in small or poorly equipped hospitals. Missing data on fewer than five variables are allowed in our web-based calculator, and the background can still provide a prediction based on ML imputation methods. However, to take full advantage of our model, we recommend that all required features be collected.

Another notable advantage of our model is that it balances the accuracy and simplicity. To enhance clinical utility, translating a complex model into a simple measure that can be readily applied by clinicians is needed. However, in previous studies, simplicity has always come at the expense of accuracy. The presentations of existing models mainly comprise sum scores and nomograms, and their simplicity is achieved by dichotomizing continuous variables, simplifying the non-linearity to a linear relationship, or reducing the equations to simple scores, which undoubtedly leads to the loss of much prognostic potential of available information.^3^ Furthermore, these models are unable to handle the complex interactions between multiple features that are characteristic of real-world clinical problems, as they are derived from conventional statistical methods. Existing ML-based models, although they solve the above methodological problems and generate high performance, do not have any type of model presentation, and the lack of presentation largely hampers their use in clinical practice.^7–21^ Instead of relying on simpler models (for example, logistic), in our study, we store the finalized intact ML model on a cloud-computing server, develop an application programming interface to access our models, and scale the platform to provide instant and automatic prediction of a patient’s outcome. This will largely improve accessibility and the real-world application of our model while retaining its high discrimination performance.

We also combined complex ML models with intuitive explanations to make them more reliable and transparent. Some ML methodologies are opaque; in other words, it may not be possible to verify how they arrive at their conclusions, which can, as a consequence, affect clinicians’ confidence when applying ML-based technologies in clinical decision-making. The explanations obtained from he SHAP analysis revealed clinically meaningful insights about the contributions of various predictors and reasoning processes behind the model’s decisions, allowing clinicians to understand the internal logic of ML and thus helping to remove the ‘trust barrier’ between users and ML models. It also provided valuable and detailed information tailored to an individual patient, potentially driving targeted interventions in clinical practice to improve outcomes.

Our results suggest that the PFCML-MT score could be of clinical importance for several reasons. First, the model output can be used to inform and discuss with patients and their relatives the prognosis following MT and help to set realistic expectations. Second, accurately predicting long-term prognosis can be of benefit even for patients with poor prognosis who already underwent MT because treatment options beyond current reperfusion interventions, such as neuroprotection, multidisciplinary approach, and rehabilitation therapies, might further improve their outcomes. Third, in this study, we evaluated the relative importance of predictors and found that postoperative laboratory indices are important predictors of outcome in patients with AIS after MT. It is conceivable that managing blood glucose, improving liver and kidney function, and reducing inflammation after MT may have the greatest potential to further improve prognosis. As these factors are potentially modifiable, this requires a further randomized study.

Inevitably, our study has some limitations. First, this study was a single-center, retrospective design, and the sample size was relatively small, which might limit the generalizability of the results. Therefore, prospective studies are necessary, and replication in large studies comprising multiple centers are greatly needed for implementing this tool in clinical settings. Second, the CT features used in this study were obtained from the first CT scan. Follow-up CT scans may provide more information, and deep learning or radiomics approaches may also increase prognostic performance. Third, although a number of potential features were analyzed, we could not fully exclude the possibility that other unmeasured or residual variables would have further improved prognosis identification.

## Conclusions

In conclusion, the PFCML-MT validated models, built on ML techniques and routinely available clinical parameters, can be accessed via the web and serve as a simple rapid estimate of prognosis for patients with AIS treated with MT. We believe that with further development and validation, this prediction model has the potential for the early prediction of prognosis, thus offering an appropriate complement to clinical judgment, supporting tailored treatment and follow-up plans, and establishing sensible treatment expectations.

10.1136/jnis-2022-019598.supp2 Supplementary data

## Data Availability

Data are available upon reasonable request.
