# Supplementary material for: An explainable machine learning model for predicting the outcome of ischemic stroke after mechanical thrombectomy
Source: J Neurointerv Surg. 2022 Nov 29;15(11):1136–41. doi: 10.1136/jnis-2022-019598 (PMC10579503; doi:10.1136/jnis-2022-019598)

Supplementary Material

Supplementary methods

Imaging analysis

Standardized multiparametric neuroimaging, including non-contrast head CT (NCCT), CT angiography (CTA) and CT perfusion (CTP), was performed in the emergency room using a 64-slice CT device (Discovery CT750 HD, GE Healthcare, Milwaukee, WI, USA) when patients arrived at the hospital.

The extent of early ischaemic changes was assessed on baseline NCCT using the Alberta Stroke Program Early CT Score (ASPECTS) methodology.10 Collateral status was determined on maximum intensity projection (MIP) images of CTA using the modified Tan score: good collateral status was defined as collaterals filling ≥50% of the occluded arterial territory, whereas poor collaterals filled <50% of the occluded territory.[1] As described elsewhere, CTP data were analysed automatically by commercially available F-STROKE Software (NeuroBlem, Ltd. Co., version 1.0.7) to generate maps of time to maximum (Tmax), relative cerebral blood volume (rCBV), and relative cerebral blood flow (rCBF) index.[2–3]

References:

1 Tan JC, Dillon WP, Liu S, *et al*. Systematic comparison of perfusion-CT and CT-angiography in acute stroke patients. *Ann Neurol* 2007;61: 533-543.

2 Shi Z, Li J, Zhao M, *et al*. Baseline Cerebral Ischemic Core Quantified by Different Automatic Software and Its Predictive Value for Clinical Outcome. *Front Neurosci* 2021;15: 608799.

3 Wang C, Shi Z, Yang M, *et al*. Deep learning-based identification of acute ischemic core and deficit from non-contrast CT and CTA. *J Cereb Blood Flow Metab* 2021;41: 3028-3038.

Supplementary table 1 List of potential candidate predictors

Category	Number of variables	Variables
Demographics	2	Age
		Gender
Stroke characteristics	4	Baseline NIHSS score
		Baseline mRS score
		Site of occlusion
		Etiology based on TOAST
Comorbidities and	10	Hypertension

lifestyle factors		Diabetes
		Hyperlipidemia
		Atrial fibrillation
		Coronary disease
		History of TIA or stroke
		SBP
		DBP
		Habitual smoking
		Alcohol assumption
Radiological findings	9	ASPECTS
		Collateral status
		CBF < 30%
		Tmax > 4s
		Tmax > 6s
		Tmax > 8s
		Tmax > 10s
		Mismatch volume
		Mismatch ratio
Treatments	5	Thrombolysis with rtPA
		mTICI
		Onset-to-puncture time
		Puncture-to-reperfusion time
		Onset-to-reperfusion time
Laboratory parameters (preoperative)	23	WBC
		RBC
		Platelet
		Neutrophils
		Monocyte
		Lymphocyte
		NLR
		Hemoglobin
		Serum creatinine
		eGFR
		Serum glucose
		Total protein
		Albumin
		Globulin
		AGR
		BUN
		Serum uric acid
		PT
		APTT
		TT
		INR

	Fibrinogen
	D-dimer
Laboratory parameters 31 (postoperative)	WBC
	RBC
	Platelet
	Neutrophils
	Monocyte
	Lymphocyte
	NLR
	Hemoglobin
	Serum creatinine
	eGFR
	Serum glucose
	Total protein
	Albumin
	Globulin
	AGR
	Cholesterol
	Triglycerides
	LDL
	HDL
	Apolipoprotein A
	Apolipoprotein B
	BUN
	Serum uric acid
	PT
	APTT
	TT
	INR
	Fibrinogen
	D-dimer
	CRP
	Hemoglobin A1c

AGR indicates albumin-to-globulin ratio; APTT, activated partial thromboplastin time; ASPECTS, Alberta Stroke Program Early CT Score; BUN, blood urea nitrogen; CBF, cerebral blood flow; CRP, C-reactive protein; DBP, diastolic blood pressure; eGFR, estimated glomerular filtration rate; HDL, low density lipoprotein; INR, international normalized ratio; LDL, low density lipoprotein; mRS, modified Rankin Scale; mTICI, modified Thrombolysis in Cerebral Infarction; NIHSS, National Institutes of Health Stroke Scale; NLR, neutrophil-to-lymphocyte ratio; PT, prothrombin time; RBC, red blood cell; rtPA, tissue-type plasminogen activator; SBP, systolic blood pressure; TIA, transient ischemic attack; Tmax, time to maximum; TOAST, Etiology based on TOAS; TT, thromboplastin time; WBC, white blood cell.

Supplementary table 2 Mathematical algorithm

Algorithm	Parameters
Base model	
AdaBoost	AdaBoostClassifier(algorithm='SAMME', base_estimator=DecisionTreeClassifier(ccp_alpha=0.0, class_weight='balanced', criterion='gini', max_depth=3, max_features=0.6227762928159657, max_leaf_nodes=None, min_impurity_decrease=0.00033005619051851146, min_impurity_split=None, min_samples_leaf=3, min_samples_split=3, min_weight_fraction_leaf=0.0, presort='deprecated', random_state=25, splitter='best'), learning_rate=0.0075783178873011225, n_estimators=268, random_state=25)
LightGBM	LGBMClassifier(bagging_fraction=0.5418445065929811, bagging_freq=2, boosting_type='gbdt', class_weight='balanced', colsample_bytree=1.0, feature_fraction=0.8152051323709875, importance_type='split', learning_rate=1.3925872044357878e-06, max_depth=-1, min_child_samples=5, min_child_weight=0.001, min_split_gain=0.5190237999874658, n_estimators=145, n_jobs=-1, num_leaves=122, objective=None, random_state=25, reg_alpha=1.0952088538329306e-08, reg_lambda=0.0044953289196024235, silent='warn', subsample=1.0, subsample_for_bin=200000, subsample_freq=0)

XGBoost	XGBClassifier(base_score=0.5, booster='gbtree', colsample_bylevel=1,<br>colsample_bynode=1, colsample_bytree=0.704070548926186,<br>enable_categorical=False, gamma=0, gpu_id=-1,<br>importance_type=None, interaction_constraints='',<br>learning_rate=0.0010865433383343915, max_delta_step=0,<br>max_depth=2, min_child_weight=4, missing=nan,<br>monotone_constraints='()', n_estimators=257, n_jobs=-1,<br>num_parallel_tree=1, objective='binary:logistic',<br>predictor='auto', random_state=25,<br>reg_alpha=0.0001441685333959445, reg_lambda=7.083575404283483e-07,<br>scale_pos_weight=2.881820460115147, subsample=0.3914144052351526,<br>tree_method='auto', use_label_encoder=True, validate_parameters=1,<br>verbosity=0)
Gradient Boosting	GradientBoostingClassifier(ccp_alpha=0.0, criterion='friedman_mse', init=None,<br>learning_rate=0.1, loss='deviance', max_depth=3,<br>max_features=None, max_leaf_nodes=None,<br>min_impurity_decrease=0.0, min_impurity_split=None,<br>min_samples_leaf=1, min_samples_split=2,<br>min_weight_fraction_leaf=0.0, n_estimators=100,<br>n_iter_no_change=None, presort='deprecated',<br>random_state=25, subsample=1.0, tol=0.0001,<br>validation_fraction=0.1, verbose=0,<br>warm_start=False)
Extra trees	ExtraTreesClassifier(bootstrap=True, ccp_alpha=0.0,<br>class_weight='balanced_subsample', criterion='entropy',<br>max_depth=4, max_features=0.5072151903339681,<br>max_leaf_nodes=None, max_samples=None,<br>min_impurity_decrease=4.475863025707312e-09,<br>min_impurity_split=None, min_samples_leaf=2,

	min_samples_split=3, min_weight_fraction_leaf=0.0,<br>n_estimators=154, n_jobs=-1, oob_score=False,<br>random_state=25, verbose=0, warm_start=False)
Random forest	RandomForestClassifier(bootstrap=True, ccp_alpha=0.0, class_weight='balanced',<br>criterion='entropy', max_depth=7,<br>max_features=0.5718824629846555, max_leaf_nodes=None,<br>max_samples=None,<br>min_impurity_decrease=3.8484700829623946e-08,<br>min_impurity_split=None, min_samples_leaf=3,<br>min_samples_split=7, min_weight_fraction_leaf=0.0,<br>n_estimators=121, n_jobs=-1, oob_score=False,<br>random_state=25, verbose=0, warm_start=False)
CatBoost	CategoricalBoosting.Classifier(nan_mod=Min, eval_metric=Logloss, Iterations=252,<br>sampling_frequency=PerTree, leaf_estimation_method=Newton,<br>grow_policy=SymmetricTree, penalties_coefficient=1,<br>boosting_type=Plain, model_shrink_mode=Constant,<br>feature_border_type=GreedyLogSum, bayesian_matrix_reg=0.10000000149011612,<br>force_unit_auto_pair_weights=False, l2_leaf_reg=2,<br>random_strength=0.6800507307052612, rsm=1,<br>boost_from_average=False, model_size_reg=0.5,<br>pool_metainfo_options={'tags': {}}, Subsample=0.800000011920929,<br>use_best_model=False, class_names=[0, 1],<br>random_seed=25, depth=5,<br>posterior_sampling=False, border_count=254,<br>class_weights=[1, 2.0810811519622803],<br>classes_count=0,auto_class_weights=Balanced, sparse_features_conflict_fraction=0,<br>leaf_estimation_backtracking =AnyImprovement,<br>best_model_min_trees=1, model_shrink_rate=0,<br>min_data_in_leaf=1, loss_function=Logloss,

	learning_rate=0.0010865433141589165, score_function=Cosine, task_type=CPU, leaf_estimation_iterations=10, bootstrap_type=MVS, max_leaves=32)
Final model	
PFCML-MT	RandomForestClassifier(bootstrap=True, ccp_alpha=0.0, class_weight='balanced', criterion='entropy', max_depth=7, max_features=0.5718824629846555, max_leaf_nodes=None, max_samples=None, min_impurity_decrease=3.8484700829623946e-08, min_impurity_split=None, min_samples_leaf=3, min_samples_split=7, min_weight_fraction_leaf=0.0, n_estimators=121, n_jobs=-1, oob_score=False, random_state=25, verbose=0, warm_start=False)

AdaBoost indicates Adaptive Boosting; CatBoost, Categorical Boosting; LightGBM, Light Gradient Boosting Machine; XGBoost, eXtreme Gradient Boosting.

Supplementary table 3 Patient characteristics in the derivation cohort and temporal validation cohort

	Derivation cohort			Temporal validation cohort		
	Good outcome (n = 52)	Poor outcome (n = 111)	P value	Good outcome (n = 20)	Poor outcome (n = 34)	P value
Demographics						
Age, years	65.71 (12.65)	73.22 (10.32)	<0.001*	66.25 (9.62)	73.44 (11.00)	0.019*
Male, n(%)	29 (55.77)	58 (52.25)	0.675	12 (60.00)	19 (55.88)	0.768
Stroke characteristics						
Baseline NIHSS score	11 (9-15)	16 (13-20)	<0.001*	11 (8-14)	16 (13-20)	0.003*
Baseline mRS score	4 (3-4)	4 (4-5)	0.011*	4 (4-4)	4 (4-4)	0.961
Site of occlusion, n (%)			0.253			0.514
Internal carotid artery	13 (25.00)	42 (37.84)		9 (45.00)	13 (38.24)	

M1-middle cerebral artery	27 (51.92)	50 (45.05)		7 (35.00)	17 (50)	
M2-middle cerebral artery or other tributaries	12 (23.08)	19 (17.12)		4 (20.00)	4 (11.76)	
Etiology based on TOAST, n (%)			0.597			0.840
Large artery atherosclerosis	22 (42.31)	46 (42.99)		9 (45.00)	14 (41.18)	
Cardioembolic	23 (44.23)	52 (48.60)		10 (50.00)	16 (47.06)	
Others	7 (13.46)	9 (8.41)		1 (5.00)	4 (11.76)	
Comorbidities and lifestyle factors						
Hypertension, n (%)	26 (50.00)	82 (73.87)	0.003*	16 (80.00)	28 (82.35)	1.000
Diabetes, n (%)	8 (15.38)	41 (36.94)	0.005*	2 (10.00)	13 (39.36)	0.031*
Dyslipidemia, n (%)	18 (34.62)	36 (32.43)	0.783	9 (45.00)	10 (29.41)	0.247
Atrial fibrillation, n (%)	27 (51.92)	66 (59.46)	0.365	9 (45.00)	18 (52.94)	0.573
Coronary disease, n (%)	3 (5.77)	22 (19.82)	0.020*	3 (15.00)	5 (14.71)	1.000
History of TIA or stroke, n (%)	9 (17.31)	24 (21.62)	0.523	2 (10.00)	5 (14.71)	1.000
SBP, mmHg	139.79 (20.27)	138.84 (23.56)	0.802	131.95 (23.54)	144.41 (26.43)	0.088
DBP, mmHg	79.77 (15.09)	76.62 (14.40)	0.202	72.95 (11.05)	72.85 (17.71)	0.982
Habitual smoking, n (%)	15 (28.85)	17 (15.32)	0.043*	5 (25.00)	4 (11.76)	0.266
Alcohol assumption, n (%)	12 (23.08)	12 (10.81)	0.039*	5 (25.00)	2 (5.88)	0.087
Radiological findings						
ASPECTS	9 (8-10)	8 (8-10)	0.112	8 (8-9)	9 (8-9)	0.659
Good collateral status, n (%)	36 (75.00)	29 (28.71)	<0.001*	12 (63.16)	13 (41.94)	0.145
CBF < 30%, ml	0 (0-11.35)	6.90 (0-34.90)	0.006*	6.60 (0-41.50)	11.30 (0.68-39.73)	0.432
Tmax > 4s, ml	240.40 (173.85-337.25)	300.10 (193.20-463.70)	0.085	307.10 (211.60-371.10)	286.05 (224.85-381.60)	0.790
Tmax > 6s, ml	129.05 (83.63-174.48)	162.20 (101.10-219.40)	0.045*	155.08 (96.52)	162.87 (118.45)	0.811
Tmax > 8s, ml	98.20 (46.45-145.15)	132.20 (64.30-190.80)	0.048*	94.58 (76.63)	77.97 (14.24)	0.960
Tmax > 10s, ml	44.35 (7.38-72.43)	61.70 (15.20-116.30)	0.018*	34.00 (5.40-131.80)	36.90 (6.90-93.88)	0.829
Mismatch volume, ml	114.95 (81.60-168.88)	127.40 (84.20-185.90)	0.312	137.71 (81.57)	136.12 (105.28)	0.955
Mismatch ratio†	0 (0-0.055)	0.064 (0-0.207)	0.003*	0.058 (0-0.173)	0.079 (0.005-0.256)	0.326
Treatments						
Thrombolysis with rtPA, n (%)	18 (34.62)	41 (36.94)	0.774	8 (40.00)	12 (35.29)	0.729

mTICI (2b-3), n (%)	50 (96.15)	95 (86.36)	0.058	20 (100.00)	30 (88.24)	0.285
Onset-to-puncture time, min	415.00 (270.00-555.75)	350.00 (240.00-550.00)	0.248	335.00 (263.00-720.00)	420.00 (310.00-570.00)	0.414
Puncture-to-reperfusion time, min	80 (60-103.75)	110.00 (90.00-150.00)	<0.001*	113.50 (35.14)	113.09 (71.40)	0.981
Onset-to-reperfusion time, min	492.50 (340.00-638.00)	470.00 (350.00-705.00)	0.839	450.00 (369.00-768.75)	480.00 (390.00-607.50)	0.738
Laboratory parameters (preoperative)						
WBC, × 10⁹ /L	6.70 (5.38-8.43)	7.90 (6.68-9.60)	0.001*	7.65 (6.00-8.55)	8.25 (6.63-9.95)	0.184
RBC, × 10^{12} /L	4.37 (0.50)	4.45 (0.54)	0.486	4.66 (0.59)	4.19 (0.64)	0.016*
Platelet, × 10^9 /L	154.50 (121.75-204.00)	191.00 (153.50-234.50)	0.002*	198.00 (133.00-241.75)	196.00 (145.50-231.50)	0.753
Neutrophils, × 10^9 /L	4.55 (3.78-6.13)	5.45 (4.28-7.73)	0.046*	5.80 (3.37)	7.13 (3.55)	0.212
Monocyte, × 10^9 /L	0.50 (0.40-0.60)	0.50 (0.40-0.70)	0.013*	0.59 (0.50)	0.53 (0.16)	0.500
Lymphocyte, × 10^9 /L	1.40 (1.00-1.70)	1.50 (1.10-2.20)	0.056	1.30 (0.90-2.33)	1.25 (0.90-1.68)	0.378
NLR	3.24 (2.82-5.38)	3.89 (1.92-6.36)	0.991	4.38 (2.85)	6.61 (4.59)	0.073
Hemoglobin, g/L	136.22 (16.52)	136.44 (18.76)	0.952	138.44 (13.61)	127.89 (19.24)	0.049*
Serum creatinine, umol/L	64.50 (56.35-72.18)	73.95 (61.23-95.25)	0.001*	66.65 (55.88-83.58)	69.55 (46.63-78.28)	0.848
eGFR, mL/min/1.73 m²	93.33 (17.28)	77.27 (22.90)	<0.001*	88.07 (20.24)	86.75 (23.28)	0.844
Serum glucose, mmol/L	6.50 (5.75-7.85)	7.30 (5.95-9.00)	0.033*	6.60 (6.00-7.88)	8.20 (6.40-10.90)	0.138
Total protein, g/L	71.26 (7.50)	73.90 (6.89)	0.101	75.44 (6.22)	70.65 (5.90)	0.021*
Albumin , g/L	41.01 (3.83)	41.07 (4.21)	0.943	42.51 (3.25)	39.78 (3.45)	0.019*
Globulin, g/L	30.26 (4.62)	32.83 (4.71)	0.016*	32.93 (5.01)	30.46 (4.73)	0.130
AGR	1.38 (0.17)	1.27 (0.20)	0.023*	1.32 (0.20)	1.31 (0.19)	0.882
BUN, umol/L	5.20 (4.21-6.40)	6.18 (5.03-7.66)	0.006*	6.13 (4.40-7.31)	5.95 (5.40-6.49)	0.963
Serum uric acid, umol/L	307.09 (78.97)	356.11 (92.17)	0.003*	372.39 (126.97)	324.85 (118.44)	0.207
PT, s	12.00 (11.30-12.60)	11.90 (11.10-12.40)	0.307	12.30 (11.60-13.03)	12.10 (11.60-12.75)	0.535
APTT, s	26.20 (24.90-27.75)	26.20 (24.90-27.50)	0.853	25.55 (24.28-26.65)	24.90 (23.85-26.90)	0.386
TT, s	18.50 (17.65-19.20)	18.40 (17.30-19.30)	0.537	17.70 (17.25-18.38)	17.50 (16.80-18.08)	0.471
INR	1.05 (0.99-1.11)	1.04 (0.97-1.09)	0.242	1.07 (1.02-1.14)	1.05 (1.01-1.12)	0.491
Fibrinogen, g/L	2.70 (2.35-3.00)	2.90 (2.40-3.60)	0.014*	2.95 (2.48-3.53)	2.75 (2.43-3.93)	0.795
D-dimer, mg/L	0.51 (0.32-1.77)	0.89 (0.50-2.17)	0.017*	0.43 (0.25-1.93)	1.24 (0.62-3.28)	0.029*
Laboratory parameters (postoperative)						
WBC, × 10^9 /L	8.00 (6.00-9.80)	10.20 (8.10-11.95)	<0.001*	8.78 (3.25)	10.19 (3.31)	0.134
RBC, × 10^{12} /L	4.10 (0.54)	4.03 (0.53)	0.430	4.08 (3.66-4.66)	3.85 (3.57-4.41)	0.179
Platelet, × 10^9 /L	162.69 (57.02)	183.26 (62.34)	0.047*	172.00 (142.25-210.00)	181.00 (137.50-233.50)	0.788
Neutrophils, × 10^9 /L	6.60 (4.70-8.00)	8.20 (6.50-10.40)	<0.001*	6.50 (5.33-8.23)	8.55 (6.78-11.08)	0.026*

Monocyte, × 10^9 /L	0.40 (0.30-0.60)	0.50 (0.40-0.70)	0.012*	0.40 (0.30-0.58)	0.50 (0.38-0.63)	0.214
Lymphocyte, × 10^9 /L	1.00 (0.70-1.40)	0.90 (0.60-1.30)	0.328	0.90 (0.70-1.30)	0.80 (0.50-1.00)	0.059
NLR	6.14 (4.56-9.00)	8.00 (6.01-16.00)	<0.001*	6.58 (4.90-10.94)	11.46 (6.53-17.44)	0.005*
Hemoglobin, g/L	127.02 (18.92)	124.09 (18.93)	0.366	125.60 (15.70)	116.88 (20.27)	0.105
Serum creatinine, umol/L	63.00 (53.00-70.00)	73.50 (60.00-92.25)	<0.001*	63.00 (59.00-70.50)	73.00 (52.50-81.00)	0.259
eGFR, mL/min/1.73 m^2	94.88 (88.14-106.61)	81.19 (63.21-93.80)	<0.001*	99.44 (88.81-113.20)	86.97 (72.35-102.23)	0.017*
Serum glucose, mmol/L	5.39 (4.73-6.70)	6.97 (5.83-9.40)	<0.001*	6.07 (5.31-7.19)	7.51 (6.12-12.58)	0.009*
Total protein, g/L	62.69 (4.99)	62.28 (5.51)	0.650	62.93 (5.98)	62.16 (4.72)	0.608
Albumin, g/L	38.93 (2.23)	37.03 (3.02)	<0.001*	38.57 (3.20)	37.67 (3.62)	0.365
Globulin, g/L	23.77 (3.88)	25.25 (4.00)	0.029*	23.15 (20.60-28.25)	24.10 (21.75-25.95)	0.707
AGR	1.67 (0.25)	1.50 (0.25)	<0.001*	1.62 (0.25)	1.57 (0.27)	0.500
Cholesterol, mmol/L	4.27 (0.95)	4.08 (1.18)	0.314	4.63 (1.34)	4.07 (0.83)	0.068
Triglycerides, mmol/L	0.92 (0.70-1.43)	0.88 (0.70-1.21)	0.284	0.96 (0.38)	1.17 (0.50)	0.113
LDL, mmol/L	2.48 (0.75)	2.39 (1.03)	0.557	2.85 (1.22)	2.38 (0.71)	0.082
HDL, mmol/L	1.22 (0.34)	1.20 (0.33)	0.712	1.21 (0.99-1.39)	1.04 (0.90-1.14)	0.078
Apolipoprotein A, g/L	0.97 (0.18)	0.95 (0.19)	0.524	1.02 (0.17)	0.95 (0.17)	0.161
Apolipoprotein B, g/L	0.75 (0.20)	0.71 (0.25)	0.304	0.81 (0.31)	0.71 (0.20)	0.133
BUN, umol/L	4.70 (4.05-6.10)	6.40 (4.75-7.70)	<0.001*	5.15 (3.73-7.45)	5.80 (5.00-7.05)	0.157
Serum uric acid, umol/L	315.33 (88.09)	348.55 (91.38)	0.030*	329.50 (90.95)	333.42 (100.31)	0.887
PT, s	11.55 (11.10-12.20)	11.60 (11.10-12.40)	0.231	11.70 (11.00-12.60)	11.70 (11.05-12.20)	0.864
APTT, s	25.95 (24.88-27.38)	26.10 (25.10-27.30)	0.736	26.40 (25.20-27.60)	25.20 (23.55-26.75)	0.067
TT, s	18.45 (17.38-19.93)	17.90 (17.00-19.10)	0.090	17.80 (16.80-19.60)	17.30 (16.55-17.95)	0.047*
INR	1.01 (0.97-1.07)	1.02 (0.97-1.09)	0.440	1.03 (0.96-1.11)	1.03 (0.97-1.07)	0.985
Fibrinogen, g/L	2.59 (0.72)	2.91 (0.87)	0.025*	2.10 (1.90-3.00)	2.90 (2.30-3.50)	0.044*
D-dimer, mg/L	1.32 (0.70-2.67)	2.45 (1.43-5.44)	<0.001*	1.74 (0.63-4.17)	3.02 (1.69-4.79)	0.074
CRP, mg/L	4.80 (2.90-10.48)	9.40 (4.70-23.40)	<0.001*	4.70 (2.40-9.20)	11.10 (5.25-23.95)	0.039*
Hemoglobin A1c, %	5.75 (5.50-6.23)	5.90 (5.60-6.90)	0.040*	5.80 (5.53-6.18)	6.10 (5.73-7.45)	0.033*

AGR indicates albumin-to-globulin ratio; APTT, activated partial thromboplastin time; ASPECTS, Alberta Stroke Program Early CT Score; BUN, blood urea nitrogen; CBF, cerebral blood flow; CRP, C-reactive protein; DBP, diastolic blood pressure; eGFR, estimated glomerular filtration rate; HDL, low density lipoprotein; INR, international normalized ratio; LDL, low density lipoprotein; mRS, modified Rankin Scale; mTICI, modified Thrombolysis in Cerebral Infarction; NIHSS, National Institutes of Health Stroke Scale; NLR, neutrophil-to-lymphocyte ratio; PT, prothrombin time; RBC, red blood cell; rtPA, tissue-type plasminogen activator; SBP, systolic blood pressure; TIA, transient ischemic attack; Tmax, time to maximum; TOAST, Etiology based on TOAS; TT,

thromboplastin time; WBC, white blood cell.

* $p < 0.05$.

† Since ischeamic core volume (CBF < 30%) in a small number of patients is 0 ml, here the mismatch ratio was defined as volume $_{CBF< 30\%}$ / volume $_{Tmax >6s}$.

Supplementary figure 1 Workflow of the study

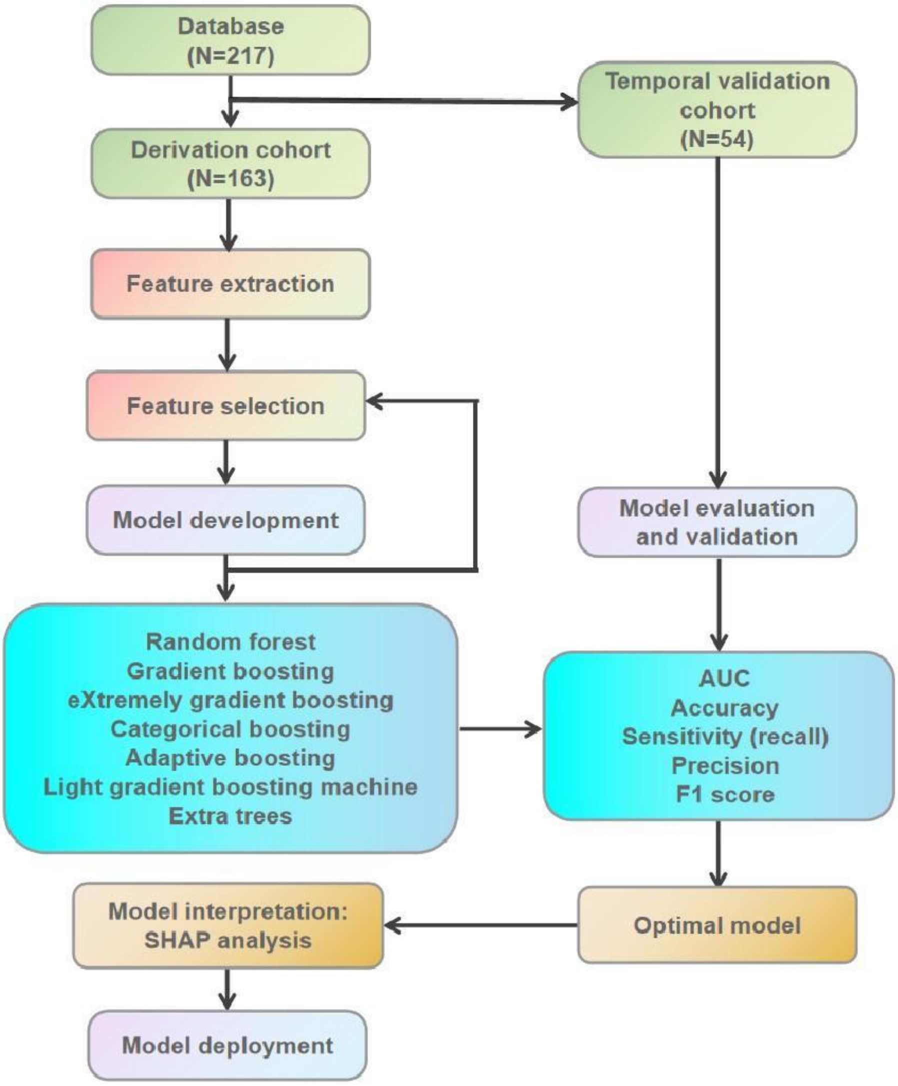

Supplementary figure 2 SHAP summary plot for the top 30 predictors contributing to the base random forest model

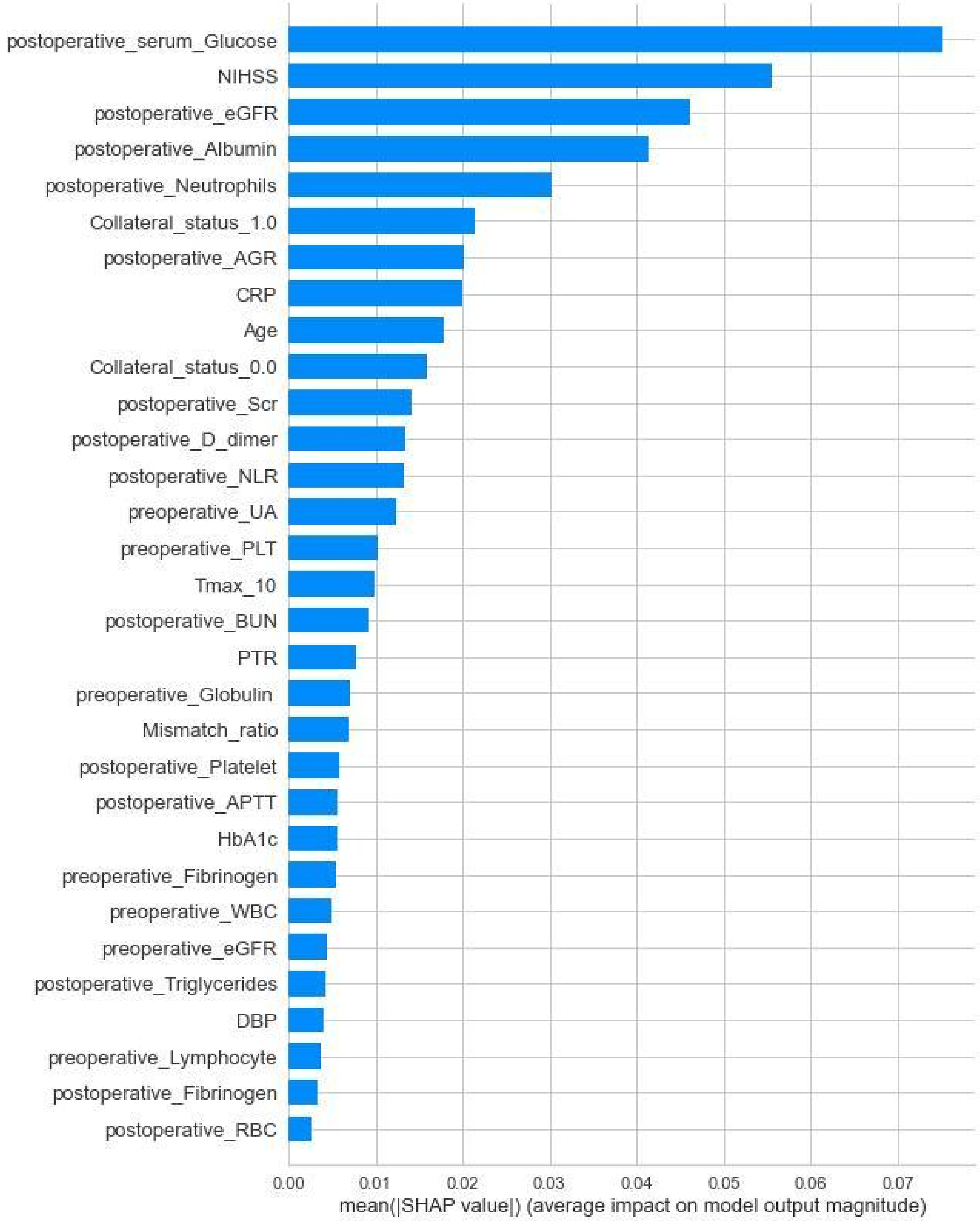

Supplementary figure 3 Area under the receiver operating characteristic curves in the test set predicting outcome based on ML models with restricted and unrestricted predictors

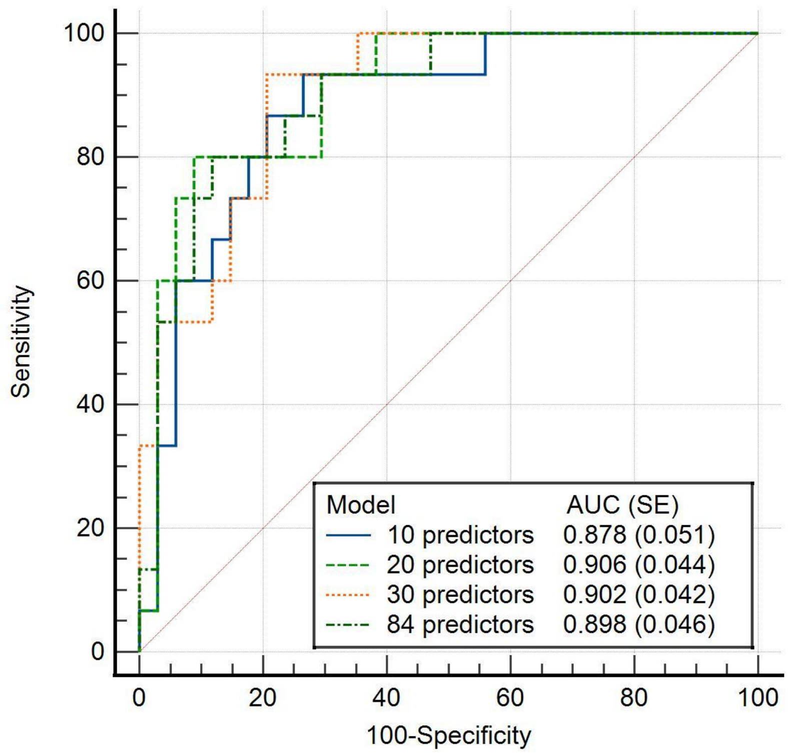

Supplementary figure 4 SHAP dependence plot of the PFCML-MT model

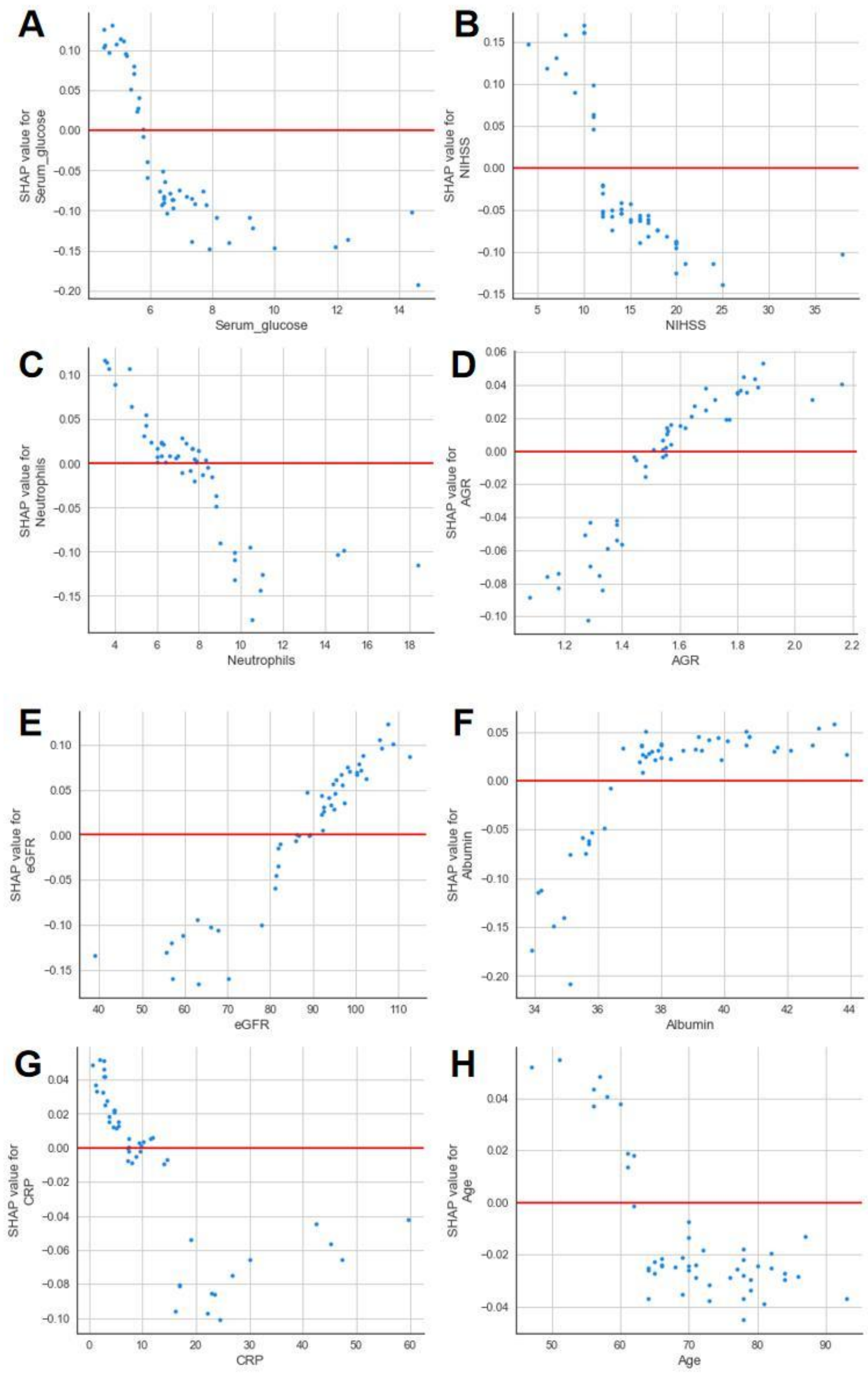

Supplementary figure 5A and 5B Force plots for 2 representative subjects with and without functional independence. The features with high impact (SHAP values) are displayed explicitly. (A) An instance with a high possibility of functional independence in which the features shown as red arrows push the odds of the instance (calculated by the prediction model) higher than the average value. (B) An instance with a low possibility of functional independence in which the features shown as blue arrows push the odds of the instance lower than the average value.

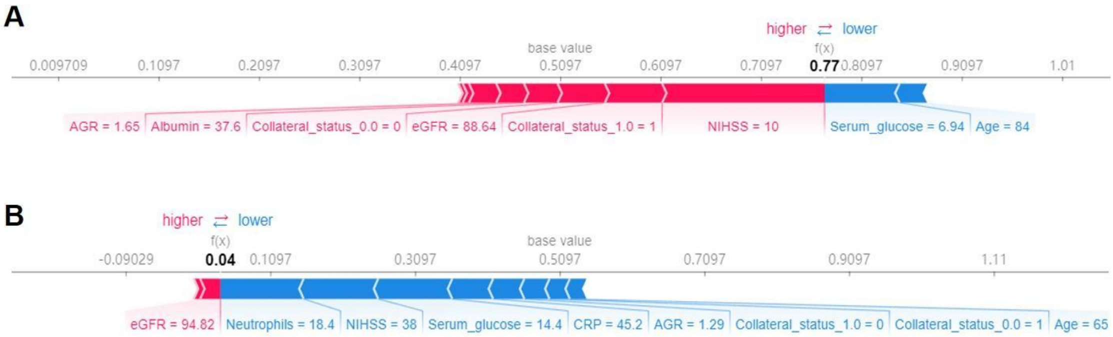

Supplement: Supplementary data [file jnis-2022-019598supp001.pdf]
